# Building IoT Services for Aging in Place Using Standard-Based IoT Platforms and Heterogeneous IoT Products

**DOI:** 10.3390/s17102311

**Published:** 2017-10-11

**Authors:** Sheik Mohammad Mostakim Fattah, Nak-Myoung Sung, Il-Yeup Ahn, Minwoo Ryu, Jaeseok Yun

**Affiliations:** 1 IoT Platform Research Center, Korea Electronics Technology Institute, Seongnam 13509, Korea; sm_fattah@yahoo.com (S.M.M.F.); nmsung@keti.re.kr (N.-M.S.); iyahn@keti.re.kr (I.-Y.A.); 2 Service Laboratory, Institute of Convergence Technology, KT R&D Center, Seoul 06763, Korea; mw.ryu@kt.com; 3 Department of Internet of Things, SCH Media Labs, Soonchunhyang University, Asan 31538, Korea

**Keywords:** Internet of Things (IoT), aging in place, standard-based IoT platforms, Web of Objects, oneM2M, virtual object, composite virtual object, service composition, medication compliance

## Abstract

An aging population and human longevity is a global trend. Many developed countries are struggling with the yearly increasing healthcare cost that dominantly affects their economy. At the same time, people living with old adults suffering from a progressive brain disorder such as Alzheimer’s disease are enduring even more stress and depression than those patients while caring for them. Accordingly, seniors’ ability to live independently and comfortably in their current home for as long as possible has been crucial to reduce the societal cost for caregiving and thus give family members peace of mind, called ‘aging in place’ (AIP). In this paper we present a way of building AIP services using standard-based IoT platforms and heterogeneous IoT products. An AIP service platform is designed and created by combining previous standard-based IoT platforms in a collaborative way. A service composition tool is also created that allows people to create AIP services in an efficient way. To show practical usability of our proposed system, we choose a service scenario for medication compliance and implement a prototype service which could give old adults medication reminder appropriately at the right time (i.e., when it is time to need to take pills) through light and speaker at home but also wrist band and smartphone even outside the home.

## 1. Introduction

The world’s population is rapidly aging. For example, the number of old people (aged 65 and over) was 562 million (8 percent of 7 billion total population) in 2012 and it increased by 55 million (to 8.5 percent of total population) in 2015, just three years later [1]. Thus, the aging population has been a global problem, which makes a large impact on government spending and economic policy in healthcare, pension, and social benefits programs—more critical in developed countries.

According to a survey in 2014, the 88 percent of the senior households in the United States would like to stay in their current residence as long as possible [2]. Thus, elderly people’s ability to live in their home independently and comfortably becomes very important because it can help reduce the social expenditure on caregiving, including time, energy, and financial resources. It would be more crucial in developed countries that have no strong public caregiving programs, for example, half of health spending (4815 USD) in the U.S. relies on private health insurance [3]. Accordingly, we need to consider technological support that allows elderly people to live in their home as long as they can care for themselves—‘age in place’ (AIP).

Technological assistance for aging in place has been developed in variety of applications [4], for example monitoring elderly parents who are living alone or disabled people, emergency response and assistance in situations that demand urgent care, helping adults with low memory capabilities recall recent actions. In particular, recent advances in information and communication technologies play a pivotal role in developing aging-in-place technologies: sensors are incorporated at home, or biological signals can directly be captured from the human body. Captured signals would be analyzed and then extracted information would be utilized to monitor physical and physiological status, infer a predefined list of emergency situations, and provide adults with emergency care services when necessary.

Due to technological advances in contactless data exchange (RFID and NFC), distributed sensor networks, short-range wireless communication (ZigBee and Bluetooth), and universal mobile access (cellular networks and WiFi hotspots), things in the world can be connected with each other, building a global network infrastructure called the Internet of Things (IoT) [5]. IoT is expected to create a huge number of innovative applications and services in our industries, in particular in healthcare and assistive living, and thus IoT will have a broad impact on our daily lives as well as the way of aging in place in an appropriate way.

In spite of such advances in IoT, there are currently two practical issues that need to be considered. The first issue concerns how to ensure interoperability of IoT systems across different industries and applications. Because many IoT products now rely on their own proprietary platforms and protocols, heterogeneous IoT systems might not be able to talk to each other and thus can’t work together. A common solution is to develop common specifications that could be deployed in developing various IoT products (e.g., pillbox and alarm devices for medication reminder services), which enables them to be interconnected and to collaborate in an efficient way. The second important issue is how people can build their new services using IoT products in an efficient way. The use of most commercial IoT products are now limited to several well-known paring services that enable people to configure an autonomous operation between two IoT products (e.g., LIFX led lightbulb in If-This-Then-That (IFTTT) [6]). However, we need an efficient way to create IoT services that leverage any IoT products we will be faced with (e.g., creating a medication alarm service which blinks LIFX led lightbulb when needed to take pills).

Our work is motivated by the issues described above and its contribution can be summarized as follows:

- Develop an aging-in-place (AIP) service platform combining previous standard IoT platforms;
- Develop a service composition tool based on standard semantic technologies;
- Develop a prototype AIP service (i.e., medication reminder service) to demonstrate how to build an AIP service using the AIP service platform and service composition tool.

The details of the proposed method are given as follows. From an extensive analysis on two types of standard IoT platforms widely used, we have developed a service platform with which a wide variety of IoT products (i.e., standard or non-standard) could be connected to each other, and their resources (i.e., name and functions) are semantically described through standard ontologies. Then, virtual objects could be automatically created, each of which represents an atomic service of the IoT products. Finally, by composing a set of virtual objects (i.e., composite virtual objects) via the composition tool, it could be possible to easily build new IoT services, e.g., medication reminder in this paper.

The remaining organization of the paper is as follows. Section 2 presents related works in AIP technologies, IoT platforms, and service composition. Section 3 introduces the system architecture based on two standard IoT platforms for building IoT services. In Section 4, we demonstrate how IoT services can be composed with virtual objects mapped from semantic description of the resources of IoT products. Section 5 illustrates our prototype IoT services for aging in place, medication reminder service. In Section 6, we discuss several limitations and remaining challenges, and Section 7 offers concluding remarks.

## 2. Background

### 2.1. Smart Home and Aging in Place

Supporting elderly or disabled people in their everyday lives has been a longstanding research area, and in particular many research activities have exploited the topic of smart aging services, which can help people age comfortably and independently while staying in their current residence as long as possible.

Chan et al. presented an extensive survey on smart homes developed around the world including U.S., Asia, Europe, Australia, and New Zealand [7]. They categorized the functions which smart homes or systems are associated with into four categories: to support, to monitor, to deliver therapy, and comfort. Through the literature survey, the authors emphasized the requirement of a variety of sensing systems incorporated into wearable or implantable devices (i.e., sensor-embedded houses) for monitoring people without intervening their daily routines. They also highlighted that assistive and interactive robots could not only provide people with useful services in their everyday lives but also serve as companions to reduce loneliness and social isolation. They extended their survey works to presents features and perspectives of smart homes in more detail [8].

Botsis and Hartvigsen introduced a literature survey (485 papers published between 1990 and 2007) on home telecare for elderly patients who suffer from chronical diseases [9]. The review results show that videoconferencing and messaging/monitoring devices could help healthcare professionals and family members reduce costs due to time savings and avoidance travelling. However, the authors highlighted that a combination of home telecare with conventional healthcare delivery is the most preferred way in telecare for elderly people.

Kleinberger et al. introduced an Assisted Living Laboratory where elderly or disabled people could be trained to handle modern interfaces for their assisted living [10]. They categorized special needs of elderly or disabled people into three categories including comfort (i.e., finding things), autonomy enhancement (i.e., cooking and eating), and emergency assistance. Finally, they stressed that elderly people will be the most demanding stakeholders for technological advancements, and future solutions for ambient intelligence such as the Assisted Living Laboratory need to focus on improving usability and accessibility with user interfaces suitable for specific situations.

Allowing the elderly to live in their current residence with a reasonable level of assistance called aging in place is an active research area, and in particular many developed countries have drawn much interest to the research projects.

Dishman highlighted four promising areas (focused on by Intel’s Proactive health research group) where technologies for adaptive aging could help older adults live healthier and more productive lives: promoting healthy behaviors, early disease detection, improved treatment compliance, support for informal caregiving [11]. Home-based healthcare technologies (i.e., personal wellness system) was introduced to encourage the elderly to maintain their everyday lives and thus live independently in their homes. A case study was performed with elderly participants—one suffering from moderate-stage dementia and the other serving as her primary caregiver. To assist their activities in daily living, a prototype system was developed in the laboratory environment, where sensors and RFID (radio frequency identification) tags were embedded into objects (e.g., cups, teapot, and chairs), and infrared camera was installed to track people’s activities.

Such field studies illustrate the potential for technology-based wellness systems that not only help caregivers monitor the elderly or disabled but also assist themselves in maintaining their health and remaining independent in their residence, for example, by giving recorded voice commands in an appropriate way to assist them in meeting their needs (i.e., cooking, eating, taking medications). Accordingly, this will result in improving quality of care for elderly people and helping reduce the social burden (e.g., avoidance of travelling in emergency conditions) and economical cost of an aging population around the world.

### 2.2. IoT Platforms and Interoperability

Embedded systems and wireless sensor networks enable a variety of sensors to be incorporated into office buildings [12] or wearable devices to be attached to occupants and biological signals to be directly collected from their body [13]. Data sets collected form sensors could be utilized to infer situations and help family members or caregivers monitor or perform emergency activities for the elderly and disabled.

Recently, advances in sensing and network technologies allow physical objects in everyday lives to be connected with each other and thus data could be shared and work together in a collaborative way. This technological trend, called IoT, has been influencing applications in many industry domains [14,15] including smart home [16], smart office [17], smart farm [18], smart grid [19], and smart city [20]. Also, it would have a great impact on wellness systems and health-related services deployed in aging-in-place solutions. For example, Yun et al. proposed a standard-based service development framework using IoT systems, called TTEO (things talk to each other), where home appliances work together according to the changes in environment conditions [21].

Because future IoT systems will probably be composed of heterogeneous hardware and software, achieving a global interoperability between them should be one of critical issues for the success of IoT markets and ecosystems. To this end, various standardization activities have been actively conducted, in particular by standards development bodies (e.g., oneM2M) and industrial consortiums (e.g., open connectivity forum and AllJoyn). For example, a standard-based IoT middleware was proposed for consumer electronics [22], and a gateway-based proxy system was developed to translate message protocols and convert data structure between different IoT systems [23]. Also several interworking studies were performed based on standard IoT platforms [24]. Most of them are focused on syntactic interoperability relying on standardized resource architecture and message protocols, which enables distinct IoT systems across different industry domains to be connected each other via application programmable interfaces and data formats such as XML (Extensible Markup Language).

More recently, research on semantic interoperability has also been highlighted, which provides a high-level interoperability where IoT systems can share their data and understand in a meaningful way without prior negotiation or integration [25,26]. Such technological challenges have been driven by the concept of the Web of Things (WoT) [27], where things’ data and functions can be abstracted and accessed through Web technologies such as HTTP protocols. In particular, the WoT framework has the potential to enable worldwide interoperability across different standards-based IoT platforms using common semantically-grounded information models including semantic description, semantic discovery, semantic reasoning, and semantic validation. Using the WoT framework, services will be able to ask for queries using semantic query languages and operates in an intelligent and meaningful way.

Lanza et al. proposed a semantically interoperable federation of IoT experimentation facilities [28]. They presented a top-level ontology, named FIESTA-IoT ontology for the interoperability of the heterogeneous testbeds and IoT platforms. They also introduced prototype services for smart cities using the FIESTA-IoT Platform. Through integrated top-level ontology and FIESTA-IoT Platform, it was shown that existing heterogeneous testbeds and different IoT platforms could be federated but also IoT services could be dynamically developed using a large number of devices connected with testbeds or platforms. From the perspectives of semantics and dynamic IoT services, this work echoes our motivation into this paper, in particular, focused on standard (i.e., oneM2M-based) services.

### 2.3. Service Composition

Service composition originally stems from the concept of service-oriented architecture (SOA) [29], and represents assembly of atomic (or composite) service components.

Brønsted et al. presented a way of combining existing atomic services to create new pervasive computing services [30]. They conducted a survey analysis on service composition mechanisms with respect to specification, runtime, and deployment indicators. Finally, they identified four main goals to be supported in service composition mechanisms in pervasive computing environments: context awareness, managing contingencies, device heterogeneity, and user empowerment.

Similarly, Kalasapur et al. proposed a service composition mechanism for pervasive computing [31]. The mechanism has employed a service-oriented middleware platform called PICO (pervasive information communities organization) [32]. The simulation studies illustrate the mechanism meets well the challenges of pervasive computing, including user and resource mobility, heterogeneity, and uncertainty of involved resources.

Sheng et al. introduced an extensive survey on Web services composition in terms of standards, research prototypes, and platforms [33]. They identified main challenges to be addressed: dependable, adaptive, autonomous, and pervasive services composition, support of REST (representational state transfer) APIs (application programming interfaces), mashup, and security support.

However, all studies summarized above focused on service composition in SOA-based systems whereas IoT systems are currently developed based on resource-oriented architecture (ROA) [34]. Accordingly, we need to consider a new way of combining SOA- and ROA-based systems for service compositions. Additionally, a majority of service applications developed using IoT systems employ an abstraction layer where their resources are represented in an abstract way and accessible through REST API [35]. However, the employment of such systems would be limited in their industry domain due to proprietary systems and data silos problem, making it more difficult to create a new service using data from distinct domains but also merge two services from distinct industry domains. For example, it is not easy to combine services from smart home and smart grid if their systems are based on their own proprietary systems. Finally, considering user mobility, dynamic network organization, and frequent presence and absence of IoT devices, a more flexible way of combining services across domains will be necessary.

### 2.4. State of the Art of Aging in Place Technologies and Our Motivation

As we analyzed above, developing, providing, and employing aging-in-place services is a very high-level, complex integration work between technologies, platforms, and human studies. In order to draw the state of the art of the aging-in-place domain and present our specific motivation, we performed another review on literature published over the last three years in aging-in-place services and technologies.

Span et al. conducted a systematic review study consisting of 26 publications related to 15 IT programs that addressed a development process of an IT application involving people with dementia [36]. From the extensive literature review, they argued that people with dementia need to be involved in all development phases of supportive IT devices and applications and provide useful feedback to improve their usefulness, acceptability, and quality of life.

Peek et al. performed a qualitative field study in the Netherlands which explores how older adults' context in physical and social activities could influence the level of use of aging-in-place technologies [37]. Also, in a newly published literature [38], they discussed older adults' perspectives on independence and views on smart home technologies for independent living. Through the study, they summarized recommendations for the design and implementation of smart home technologies needed to be carefully considered by technology suppliers, policy makers, and care providers.

Elenko et al. has redefined a term—‘digital medicine’—that will have a great impact on diagnosing, preventing, monitoring, or treating a disease, condition, or syndrome [39]. The authors highlighted importance of 'digital medicine platforms' which allow patients to be connected and thus their data, experiences, and treatment could be leveraged in a clinically relevant manner.

Heinz et al. implemented and tested tools for computer-based communication activities such as Skype and daily health diary for independent leaving of older adults [40]. They found tele-healthcare technologies can be a feasible option for monitoring their well-being of older adults, but some people experienced difficulty in testing the tools.

Kim et al. summarized digital technology to enable aging in place including novel smart home technologies [41]. They particularly introduced further studies of innovative redesigning of systems that confirm the efficacy and cost-effectiveness of using digital technologies to enable aging in place. Accordingly, it is necessary to fully redesign a platform for providing aging-in-place services in order to support adults-friendly feedback system and achieve the cost-effectiveness of information and communication technologies (ICTs) for assistive or independent living in aging in place.

Caldeira et al. performed a qualitative study for investigating how the combination of external monitoring (i.e., invasive) and self-care (i.e., non-invasive) allows retired seniors to remain independent for longer [42]. They found that a collaborative care including family members, friends, health providers, and communities is crucial for seniors' health management. This motivated the need for developing a standard platform under which all the stakeholders will be able to collaborate in an effective way.

Blackman et al. conducted an extensive survey on ambient assisted living (AAL) technologies, which could be potentially used by people living with cognitive impairment [43]. They first identified 59 technologies of interest from 64 articles published after 1990 and then classified them into 11 types, including activity monitoring, alert, emergency, feedback support, health monitoring, standards, etc. In particular, they found that only three projects were classified into the 'standards' category, being done to standardize AAL systems. This implicates that standardization in aging-in-place systems is practically required to foster internationality, interoperability, and cost-effectiveness in development and deployment, which echoes our motivation into this research.

Consel et al. presented an assistive living platform to support aging in place, which addresses daily living activities, safety, and social participation [44]. The infrastructure is based on a non-standard platform connected with sensors (i.e., contact and motion detectors) and actuators (i.e., smart plugs which can measure electricity consumptions but also turn on/off a connected home appliance), and tablets as interaction devices which give feedbacks such as notification. Although the user study shows the efficacy of their platform and assisted living services such as context-aware notification, tablet-based feedback would be inappropriate for older adults suffering from mental disorders like Alzheimer's disease. Also, such a proprietary platform has limitation on interworking with other sensors and actuators to extend their services to healthcare devices and home appliances normally used in daily lives.

Aguiar et al. introduced a networked and interoperable suite of robotic furniture consisting of side table, chair, and screens, which can support aging in place [45]. The chair and table can be controlled based on user-friendly interfaces composed of hand gesture sensors embedded into chair armrests and input toggle switch. Besides the robotic furniture, they plan to connect other IoT products such as Amazon Echo, Nest thermostat, and connected lamp to improve the functionality of the proposed aging-in-place service. However, all the robotic furniture and sensing systems implemented were connected via a network of ZigBee-based module, Arduino, and personal computer (i.e., a proprietary system) on which a Java program runs, and this implicates that even such a well-designed robot system for assistive living will clearly be hard to be interworked with other systems due to the lack of non-standard platforms and application programmable interfaces.

Lee and Dey developed the dwellSense sensor suite consisting of a pill pox equipped with door closure sensors, phone sensor, and coffee maker incorporated with various contact sensors, and used it to monitor whether older adults maintain their abilities to carry out various activities in daily living (i.e., medication taking, phone use, coffee making), called observations in daily living (ODLs) [46]. Their case studies indicated that reviewing the ODL data can help medical professionals refine care plans for their patients but also result in greater awareness of clinical team or informal caregivers. This style of observations in daily activities provided the motivation for this research into the practical implementation of more intelligent awareness of taking medication and reminder system by using sensor-augmented home appliances and interworking with various IoT products.

In summary, through this research we would like to tackle two important aspects of the aging-in-place domain: (1) a standardized platform for connecting heterogeneous devices and sharing data for the stakeholders related to the aging-in-place domain and (2) a service composition tool for helping medical professionals or informal caregivers such as family members or friends compose and provide aging-in-place services. This is particularly important because computing devices like tablets would be inappropriate for old adults having mental disorder when giving alerts (e.g., medication reminder). Instead, simple color changes of room light or playing voice command would be much more effective for assisting older adults’ behaviors in everyday lives. Accordingly, it would be necessary to provide a computer-based tool which enables informal caregivers (with little knowledge on computing systems but with full guidance of medical professionals) to build services involving the functions of any home IoT appliances in an efficient way.

## 3. System Architecture for Building IoT Services

### 3.1. Web of Objects (WoO) Platform Architecture

**Figure 1:**
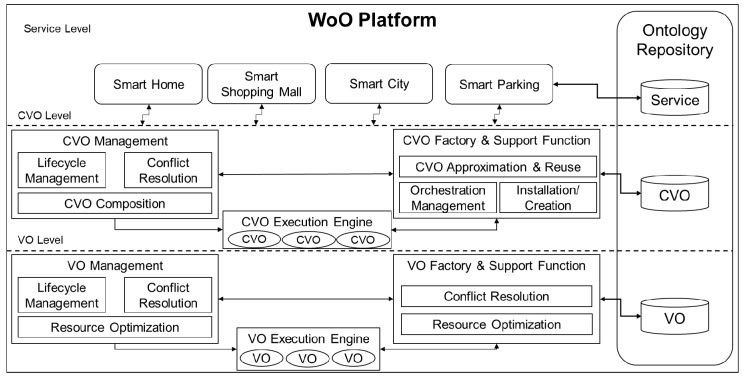
Functional overview of the Web of Objects (WoO) platform.

Web of Objects (WoO) is based on an SOA-based conceptual architecture which considers real world objects and their functionality as virtual objects (VOs) [47,48]. In WoO, VO provides atomic functionalities which are used in composition to create new composite virtual objects (CVOs). Figure 1 shows a functional architecture of the WoO platform. The VOs, at the bottom of this functional architecture, represent the virtualized real world entities. The VO management module is responsible for life cycle management, resource optimization, and conflict resolution. The VO execution engine, on the other hand, is responsible for interaction with real world entities such as sensors, actuators, etc. The VO factory and support functions handle deployment of new VOs in the WoO Platform. The idea of CVO in WoO, is adopted from cognitive management framework proposed by Vlacheas et al. [49]. According to this framework, CVOs are cognitive mashup of VOs which are semantically interoperable [50]. A CVO renders services in accordance with the application requirements and enables the reuse of existing VOs outside their initial context and domain. Any request that comes from the service level, the CVO factory and support function looks for opportunity to reuse existing CVO by matching request and situation parameters. Otherwise, it triggers creation of new CVO. New CVO is composed based on available VOs which offer relevant functionalities. The WoO platform are built on top of SOA based ideology which has some inherited advantages such as modularization, composition, model driven implementation, etc. We adopt the WoO platform in designing of our proposed system architecture because the concepts and techniques introduced by WoO platform is suitable for composition in the IoT environment. More details about WoO can be found in [51,52]. Even though we reused the concept of CVO, we redefine using standardized semantic composition language. As a result, we are able to design and implement the CVO more efficiently to be used in our proposed system. Our proposed approach of defining CVO is discussed in details in Section 4.

### 3.2. oneM2M Platform Architecture

#### 3.2.1. oneM2M Standard and Reference Model

The oneM2M project started in 2012 with the aim of creating technical specifications that address the need of a common M2M (machine to machine) and IoT service layer readily incorporated in heterogeneous hardware and software systems, enabling to develop globally applicable and access independent applications across different industry domains [53]. The oneM2M initiative is a globally joint collaboration between standard development organizations (SDOs) and industrial consortiums such as OMA (open mobile alliance) and BBF (broadband forum), where more than 200 members from industrial and research sectors work together to provide a unified framework on which different IoT-related technologies would seamlessly interconnect and interwork with each other.

**Figure 2:**
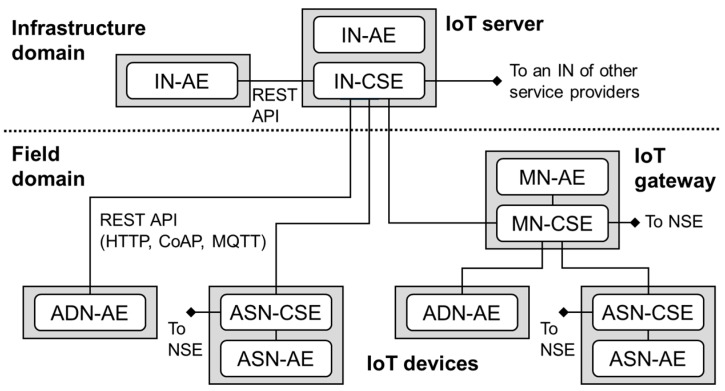
oneM2M reference architecture.

The architecture of oneM2M standards is well described in the previously published literature [54], but we here briefly introduce it for better understanding of the advantages of oneM2M standards. Figure 2 shows the oneM2M reference architecture model whose environments are divided into two domains: infrastructure and field domain. There are four types of nodes defined: infrastructure node (IN) for the infrastructure domain, middle node (MN), application service node (ASN), application dedicated node (ADN) for the field domain. The oneM2M model adopts a layered model: the application layer where application entities (AEs) are located and provide application services; the common service layer where common service entities (CSEs) are located and provide a set of common service functions (CSFs) like device and data management; and the underlying network service layer where network service entities (NSEs) are located and provide network services from the underlying network to be utilized by the CSEs.

The oneM2M reference architecture adopts the ROA model, and the resources could be uniquely addressed through REST APIs defined in the implementation of CSEs. Accordingly, given a set of predefined REST APIs and its data format (e.g., JavaScript Object Notation), all the resources located in CSEs are accessible by the basic four CRUD (create, retrieve, update, delete) operations combined with the corresponding URIs (uniform resource identifiers) on the Web-based interaction.

For interworking between oneM2M and non-oneM2M systems, the oneM2M standard defines a specialized application entity called interworking proxy entities (IPEs). The main features of an IPE are providing non-oneM2M reference points (e.g., ZigBee) to translate message protocols and convert data models to each other. Accordingly, by developing IPEs for interworking with non-oneM2M consumer products (e.g., smart bands), it could be possible to create IoT services embracing useful functionalities supported in both oneM2M and non-oneM2M products.

#### 3.2.2. oneM2M-Based Open Sources

Since the first version of oneM2M standards was released in 2015, companies and organizations have implemented their IoT platforms complying with oneM2M specifications. In particular, several nonprofit research bodies provide the open source implementation of their platforms to facilitate the widespread deployment oneM2M-based applications, including the Eclipse OM2M project [55] and IoT Data Management on OpenDayLight (ODL) project [56].

Among them, in this paper we employ open source platforms distributed by the OCEAN (Open allianCE for iot stANdards), an open source-based global partnership project for IoT standards [57]. It offers oneM2M-compliant platforms at programming code level, which enables people to develop their IoT products or applications without rich knowledge on oneM2M specifications. For our study, we employ the common IoT service platform (i.e., IN-CSE in Figure 1), called ‘Mobius’, and the software platform for IoT devices (i.e., MN, ASN, ADN in Figure 1), called ‘&Cube’. Both platforms are now available in the OCEAN developers homepage [58], and tutorials and tools are updated.

The OCEAN also provides the interworking software realizing interworking proxy entities for OCF (open connectivity foundation) [59] and LwM2M (lightweight M2M) [60], each of which allows OCF and LwM2M devices to interwork with oneM2M devices, respectively.

#### 3.2.3. oneM2M Base Ontology

**Figure 3:**
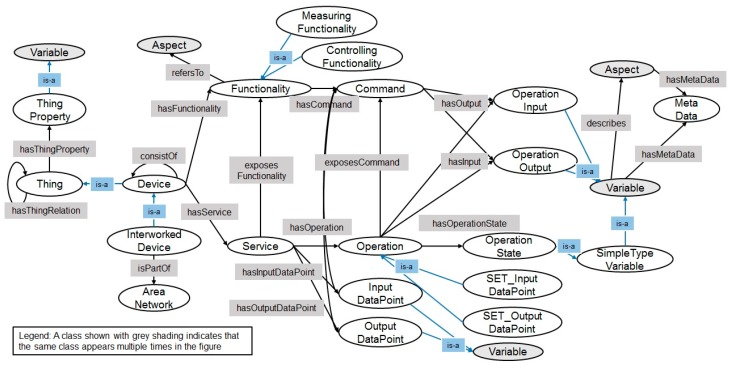
The oneM2M Base Ontology.

The oneM2M Base Ontology was developed in the oneM2M working group (i.e., MAS WG) [61]. The Base Ontology is used to provide syntactic and semantic interworking between oneM2M systems and non-oneM2M systems described by ontologies. It is designed to semantically discover entities in the oneM2M system using minimal number of classes, properties, and restrictions. Figure 3 shows the relations of classes and properties in the Base Ontology. The circles denote classes and black arrows denote object properties. 

First of all, Class:Thing is a super class that represents a device, platform, or service in the oneM2M System. Thus, it has relations with Class:ThingProperty and itself through Object Property:hasThingRelation and Object Property:hasThingProperty respectively.

Class:Device represents a device which can accomplish a particular task using its function. It also represents a device which is able to interwork with another device via network. Accordingly, Class:InterworkedDevice is a sub-class of Class:Device.

Class:Service represents functions of a device in a communication network such as remotely controllable and registerable. Hence, the representation of Class:Service depends on the network technology, and Class:Function expresses the meaning of device’s function, for example, ‘close door or open door’. The Base Ontology would describe both human- and machine-understandable perspectives with respect to a device’s function. Thus, Class:Device has the relation of Object Property:hasService between Class:Device and Class:Service and the relation of Object Property:hasFunctionality between Class:Device and Class:Functionality. In addition, a procedure-type of Class:Service and Class:Functionality are described by Class:Operation and Class:Command using Object Property:hasOperation and Object Property:hasCommand. Class:Operation represents a procedure manner in a communication network, e.g., ‘transmit data to other device.’ Class:Command describes the action command to support a function to accomplish a particular task.

At this time, each output data and input data of Class:Operation and Class:Command are represented in Class:OperationOutput and Class:OperationInput. Class:Operation and Class:Command have the relations with Class:OperationOuput and Class:OperationInput via Operation Property:hasOutput and Operation Property:hasInput, respectively.

Finally, in order to support the CRUD (create, retrieve, update, delete) operations, Class:SET_OutputDataPoint and Class:GET_InputDataPoint are designed. CREATE operation (i.e., PUT/POST) could be defined in Class:SET_OutputDataPoint and RETRIEVE operation (i.e., GET) could be defined in GET_InputDataPoint.

### 3.3. Proposed System Architecutre: Aging in Place (AIP) Service Platform

**Figure 4:**
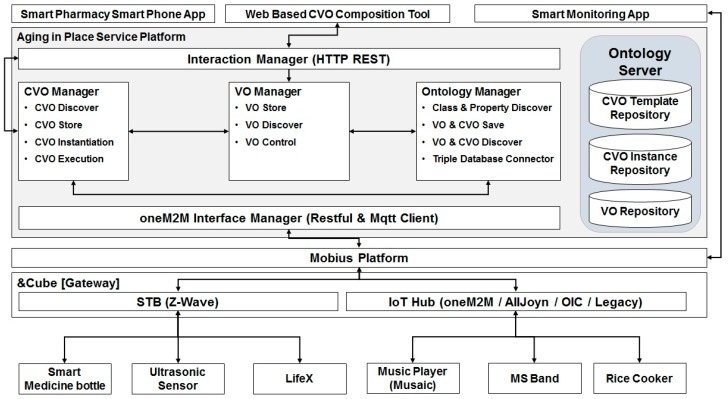
System architecture of aging in place (AIP) service platform.

Figure 4 depicts the proposed system architecture in this work. This architecture is developed by adopting the WoO platform and Mobius platform. While the Mobius is responsible for virtualizing real world objects and for providing REST APIs to interact with those entities, the WoO-based platform facilitates composition and deployment of new aging in place services. From now on, we call the integrated architecture for aging-in-place services ‘Aging in Place (AIP) service platform’.

The AIP service platform is composed of the following components: Interaction Manager, CVO Manager, VO Manager, Ontology Manager, oneM2M Interface Manager, and Ontology Server.

- **Interaction Manager:** Interaction Manager provides a suite of REST interfaces to the Web-based composition tool for CVO template creation by means of Web services. The main functionalities of this interfaces are to provide with the list of available CVOs, list of available VOs, types of VOs, and functionalities of VOs.
- **CVO Manager:** CVO Manager facilitates creation of CVO template by providing interfaces to discover and store CVO templates. It is also responsible for instantiation and execution of CVO. To store the templates and instances of CVO, it interacts with the Ontology Manager.
- **VO Manager:** VO Manager subscribes VOs in the Mobius platforms. Whenever a new VO’s semantic description is created in the Mobius, VO Manager gets a notification and stores its semantic description in the local triple database by passing it to the ontology server. Additionally, VO Manager supports the composition tool and CVO Manager by providing information about VOs.
- **Ontology Manager:** Ontology Manger uses the oneM2M base Ontology to enable creation of CVO template ontology and instance ontology and to support SPARQL queries over Ontology Server.
- **Ontology Server:** Ontology Server includes three main repositories (triple databases) which are CVO template repository, CVO instance repository, and VO repository. The data stored in these repositories are in RDF/XML format. Ontology Server provides interfaces to interact with these triple databases by means of SQARQL queries.
- **oneM2M Interface Manager:** The main responsibility of this component is to enable communication with the Mobius platform. The Mobius platform exposes its own interfaces in terms of REST Web services. As the Mobius also has an MQTT server, our AIP service platform has to include an MQTT client in this interface manager.

## 4. Service Composition Using Virtual Objects and Composite Virtual Objects

The AIP service platform is built on top of the Mobius and provides a way of composition and execution of new services for elderly people. Mobius exposes each real-world device as virtual objects along with REST and publish/subscribe APIs. Virtual objects have semantic descriptions in Mobius. When a new device is installed or new resource is deployed the oneM2M device platform, &Cube generates semantic descriptions using oneM2M annotator and stores it in Mobius as semantic descriptor of virtual objects. Each container in Mobius is considered as a virtual object. A composite virtual object combines one or more virtual objects and operations to compose new services for AIP service platform. The creation process of virtual objects and composite virtual objects are described in the following sections.

### 4.1. Virtual Objects (VOs)

A real world device or sensor is virtualized through the Mobius platform. Mobius exposes REST interfaces to retrieve information about the device or sensor. Also, using these interfaces, it is possible to send control command to the device or actuator by creation of contentInstance. When a new device (sensor or actuator) is deployed, corresponding AE and container are created in Mobius by &Cube. If new data are generated by a device, &Cube creates new contentInstance as a child node of that device’s container. Besides, if any application or user wants to send any commands to a device, a new contentInstance needs to be created under that device’s container. In this case, &Cube is notified via a subscription mechanism. Then, &Cube passes the information of contentInstance to its thing adaptation software in order to execute the command. In this work, AE represents either a particular device or a group of homogenous devices on a device platform and a container represents an atomic service provided by a particular real world entity or device. Based on this approach, we can say that a virtual object or VO in AIP service platform is represented by a combination of AE and container of the Mobius platform. In fact, this is exactly how we stored the VO description in the AIP service platform. Each VO description on the triple database is saved within a graph where the root of the graph represents the URI of a particular home of a user. This method helps us to discover and retrieve the list of VOs from the triple database easily.

**Figure 5:**
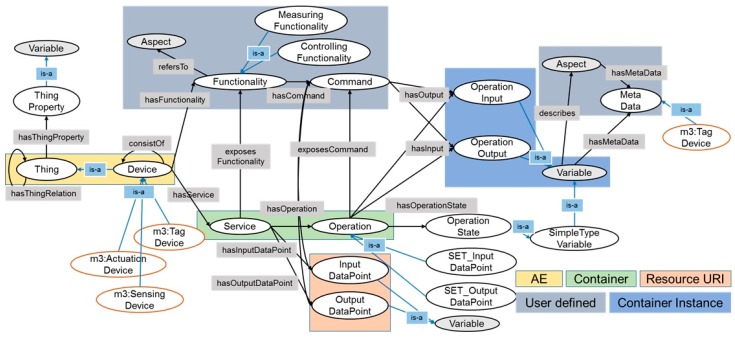
oneM2M resource mapping with oneM2M Base Ontology for Virtual Object (VO) creation.

In order to support the AIP service platform, we design and implement an oneM2M annotator to create semantic description using the oneM2M Base Ontology. The oneM2M annotator is hosted and executed on &Cube. The oneM2M annotator generates semantic description under each container. It needs a device configuration file and a device description file which are created manually during device deployment. A device configuration file contains the information of a device in Mobius. For instance, it contains an application ID, operating system, transport layer port, application protocol, host URL and port, container information, maximum limit of container instances, etc. The oneM2M annotator retrieves AE and container information from device configuration file and retrieves description about those AEs and containers from the device description file. The device description file is a mapping between oneM2M Base Ontology and Mobius resources. Figure 5 shows the mapping procedure between oneM2M resources and oneM2M Base Ontology.

**Figure 6:**
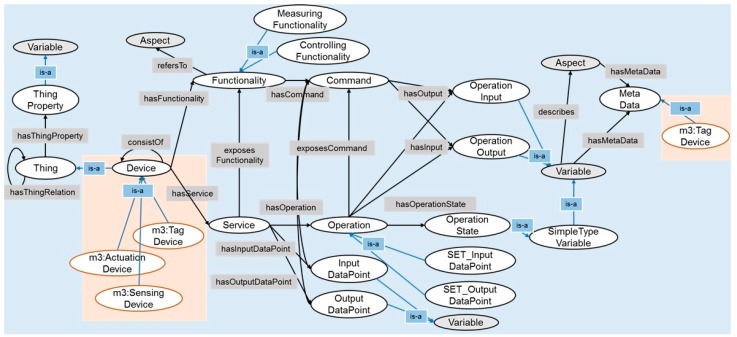
Modified oneM2M Base Ontology for VO design.

Figure 6 shows a modified oneM2M Base Ontology where M3-lite device taxonomy is defined as an equivalent class of oneM2M device class and M3-lite unite class is an equivalent class of oneM2M metadata class [62]. The reason behind using M3-lite ontology is to reuse device taxonomy and unit taxonomy which is already published in M3-lite ontology instead of defining new concepts by ourselves. Also, oneM2M standard encourages to extend oneM2M Base Ontology by using it as a super class ontology.

**Figure 7:**
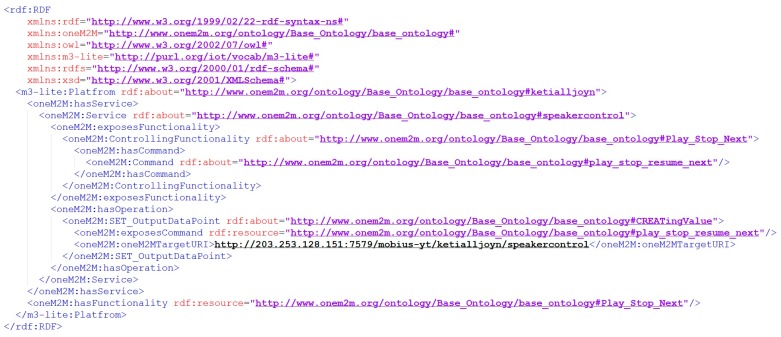
Sample oneM2M annotator generated semantic description.

The main reason behind creation of oneM2M Base Ontology based semantic description is to enable interoperability between platforms and thus service composition. We successfully represent heterogeneous devices using oneM2M Base Ontology. We also reuse them to compose CVOs where a CVO model is created using OWL-S [63]. In other words, oneM2M based semantic description is incorporated with OWL-S to enable composition. Figure 7 shows a sample description of oneM2M based semantic description of a VO generated by the oneM2M annotator.

**Figure 8:**
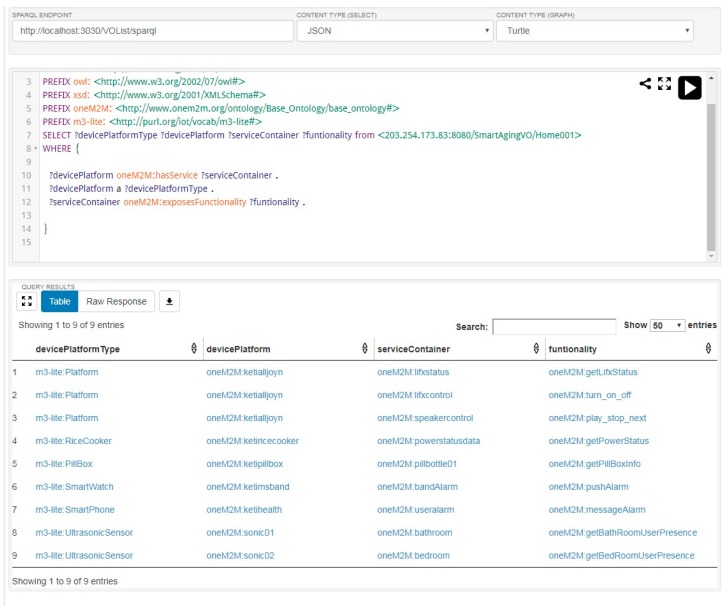
List of Virtual Objects in aging in place (AIP) service platform.

The list of VOs in the AIP service platform is shown in Figure 8. This figure shows a snapshot of running SPARQL query on Apache Jena Fuseki server [64] and the results we get from the query. In order to test and visualize the list of VOs, we host the triple database on the server and run SPARQL query. This is a flexible and user-friendly way to understand semantic triples.

### 4.2. Composite Virtual Objects (CVOs)

**Figure 9:**
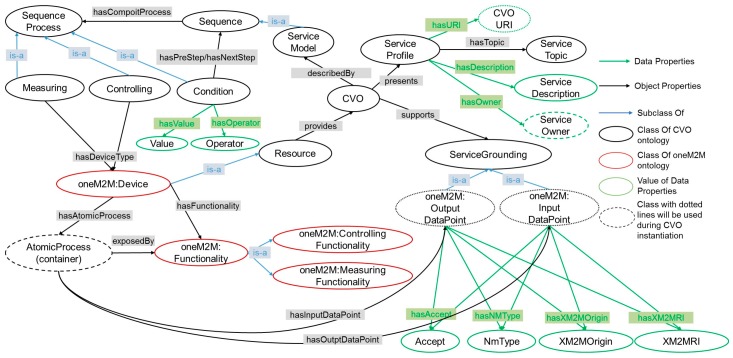
Modified OWL-S Ontology for composite Virtual Object template.

A composite virtual object or CVO in the AIP service platform comprises one or more virtual objects and a set of actions. To create a CVO, first we need a CVO template which consists of the types of VOs and set of actions. Here, actions are the functionalities that are exposed by VOs. The core of CVO template model is DAML-S service ontology and process ontology. Figure 9 illustrates a CVO template ontology model where VO ontology extends the OWL-S ontology and it is designed on protégé [65]. A CVO is equivalent to a service in the OWL-S top level service ontology. The CVO class is described by a service model which includes the OWL-S process ontology. A service model is a set of sequences where each sequence represents a composite process. The sequence process is specified by its three subclasses namely, Measuring, Controlling, and Condition. Each of the names of these subclasses reflects the functionality of each composite process. A composite process defines types of the devices which is, in this case, oneM2M device Class. If we recall our virtual object description, this class is the equivalent class of M3-lite device class. This class is mapped with oneM2M AE resource in Mobius. It also has a property called hasFuntionality through which it defines the functionality of a device. A functionality can be either controlling functionality or measuring functionality. Functionality properties are mapped with the functionality of an oneM2M container resource which is represented in the CVO ontology as an atomic process. This class is instantiated when we create a CVO from the CVO template.

A composite process can be also a conditional step where it makes a comparison and based on the comparison it goes backward or forward. A CVO class has also service profile which defines the URI of the CVO, service description, service topic, and service owner. Service grounding class is the superclass of oneM2M: Input/Output DataPoint class which is also used during instantiation of CVO to define an end point to access a particular service provided by the device. A CVO template is created based on the user request from the service composition tool.

### 4.3. Service Composition Tool

**Figure 10:**
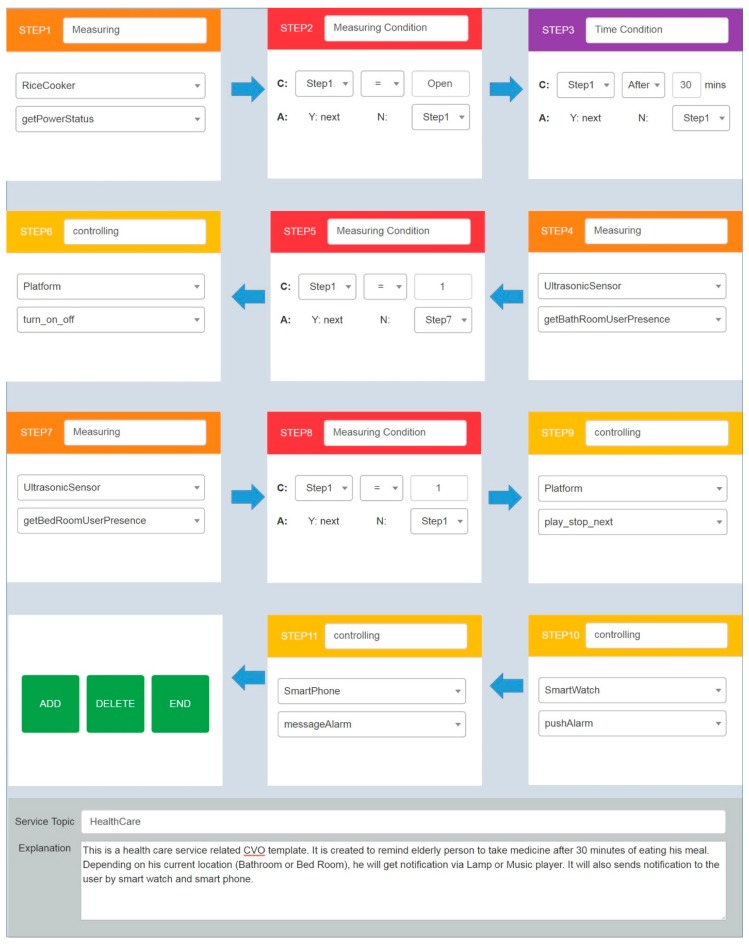
Creation of a Composite Virtual Object (CVO) for a medication reminder service.

A service composition tool is developed on top of the AIP service platform provided Web-based interfaces. The AIP service platform defines REST interfaces to retrieve the available data and functionalities of each device. Different types of CVO template can be created by using existing VO. Figure 10 shows an example of CVO template creation through the service composition tool. A user needs to define each step and corresponding functionalities to create a new CVO.

A user should first select one of seven functions in a step, which are composed of Measuring, Measuring Condition, Time Condition, Situation Condition, Emotion Condition, Controlling, and Controlling Condition. After selecting the function of a step, the user can select a device defined as an individual of the Device class of the VO corresponding to the type of functions (e.g., Measuring function and Controlling function). Finally, the AIP service platform shows available functions of the device through sematic discovery.

**Figure 11:**
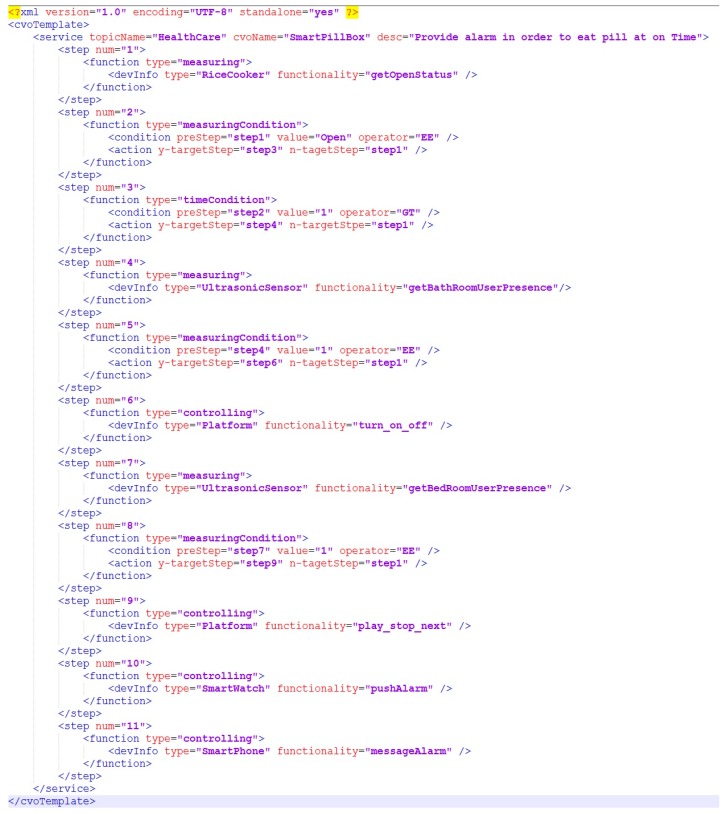
XML representation of Composite Virtual Object (CVO) template.

The example scenario describes a composite service which has 11 steps. Step 1 defines the type of the device (rice cooker) and its functionality that needs be considered for the next step. Step 2 stands for checking whether a rice cooker is open or not. We assume here if a rice cooker is opened, that implies a person has taken his meal. Step 3 defines a time condition where it checks the condition if 30 min has been elapsed after opening the rice cooker. If this condition is met, we move forward to the next step which considers the ultrasonic sensor on the bathroom. Step 5 determines whether the elderly person is in the bathroom or not. If the condition of Step 5 is met, on the Step 6, a lamp which is situated on a device platform should be turned on and off to provide signal to the elderly person on the bathroom. Similar to Steps 4 to 6, Step 7 to 9 defines that if the elderly person is in the bedroom, turn on the speaker situated on the platform. On the last two steps (Step 11 & Step 12) wrist band and smartphone will notify the person by alarm. A service composition tool generates XML data (Figure 11) and sends it to the AIP service platform. The AIP service platform creates CVO template ontology from this XML data. Afterwards, the CVO template is stored into triple database and it is retrieved during CVO instantiation. The next step of the template creation is CVO instantiation.

### 4.4. CVO Instantiation and Execution

**Figure 12:**
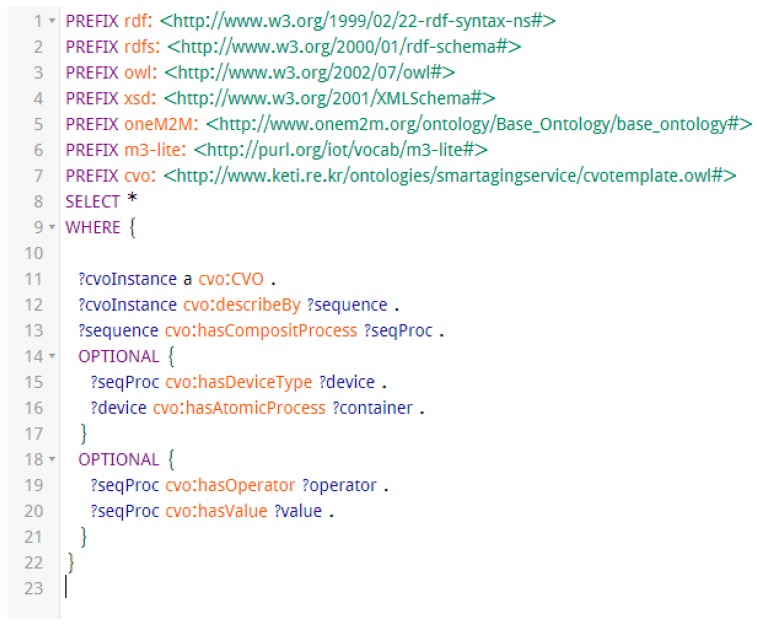
SPARQL Query to retrieve CVO Instance from the AIP service platform.

**Figure 13:**
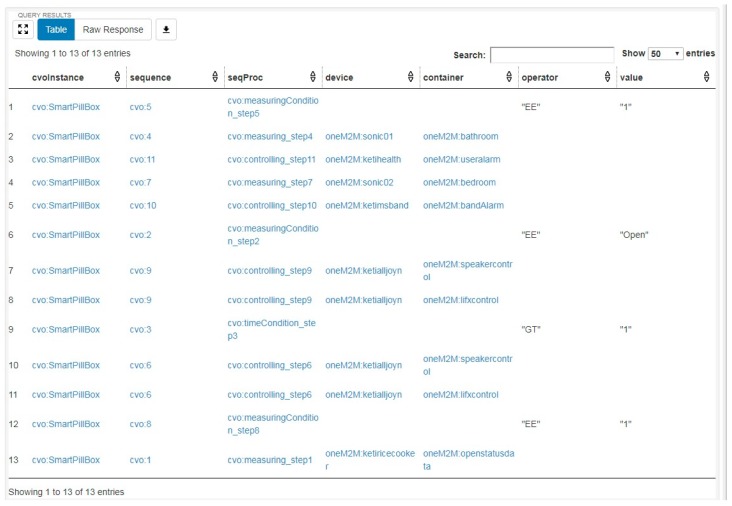
CVO Instance description result from SPARQL query.

A CVO should be instantiated from its template in order to successfully execute within the AIP service platform. During instantiation of a CVO, corresponding template is retrieved using SPARQL query from the triple store of the CVO which has been stored within the AIP service platform during template creation. A CVO instance is created based on the available device and their functionality on the home of an elderly person according to CVO template. During instantiation specific device and endpoints to interact with that device are defined. CVO instantiation also includes service owner within service profile. The description of CVO instance in our example, is generated in RDF/XML format which is not human-friendly or easily readable. In order to present the CVO instance in a simpler format we use Apache Jena Fuseki triple database server to load the CVO instance and run SPARQL query to retrieve some of the vital information about the CVO instance. Figure 12 is the query that retrieves a CVO instance from the AIP service platform along with its sequences, sequence processes, and the corresponding devices and containers (atomic process). Figure 13 displays the result from this query. The first column is same for every row which is the name of the CVO. The second column presents the sequences or steps from 1 to 11. Each of the sequences or steps has composite process called sequence process (third column) which is either measuring/controlling step or condition step. If the sequence process is about measuring or controlling a device fourth column represents the corresponding device and the fifth column stands for corresponding container.

On the other hand, if the sequence process is a conditional step then sixth and seventh columns represent corresponding operator and value. For instance, operator EE stands for equal and GT stands for greater than. The value of the operator can be string, integer, or boolean. The AIP service platform deploys a scheduler which runs once in every minute and executes the existing CVO instances on the triple store. The execution process of the CVO instances is pretty straightforward. First, the execution manager retrieves all the sequences of a particular CVO and extracts each sequence process or composite process. Each composite process is examined by the execution manager to understand whether it is a measuring/controlling step or conditional step. If the step is a measuring step, it retrieves data from the endpoint associated with the atomic process (container). If the step is a controlling step, it sends control command to the endpoint. Otherwise, it compares the condition defined in the CVO instance and moves on to the next step.

## 5. Service Development for Aging in Place

The combination of the aging-in-place service platform and composition tool enables us to build new services meeting complex customer requirements while embracing heterogeneous IoT products including non-oneM2M devices and proprietary consumer electronics.

### 5.1. Service Scenario for Aging in Place

Over past decades, many research projects in the healthcare domain have developed many kinds of application services that help elderly people maintain their life independently in their homes for as long as possible. In the process of normal aging, in particular, people may experience declines in the functional status of mental and physical activities, possibly leading to further health problems. Accordingly, researchers have worked on developing ways of evaluating health status of seniors via functional assessment.

Katz defined a list of activities necessary to live independently without help, commonly referred to Activities of Daily Living (ADL), and introduced a tool for measuring the old adults’ capacity to perform activities in daily living, called Katz index [66]. The Katz index is intended to quantitatively measure performance in basic activities required in daily living, including bathing, dressing, toileting, transferring, continence, feeding. Each index has 1 or 0 point, and thus six of total points represents high independence of the adult, otherwise very dependent as the sum point are low [67].

Similarly, Lawton and Brody extended ADL and newly identified a list of activities required to live independently in the ‘community’, commonly referred to Instrumental Activities in Daily Living (IADL) [68]. IADL is a little bit more complex than ADL and gives a measure to assess independent living skills in community. Since the first introduction in 1969, many researchers and organizations have redefined variations of IADL. But we here consider the Lawton’s instrumental activities in daily living [69], including ability to use telephone, shopping, food preparation, housekeeping, laundry, mode of transportation, responsibility for own medications, ability to handle finance.

Among all the activities listed in ADL and IADL, we chose ‘responsibility for own medications’ as the target activity. In other words, our prototype service for aging in place is designed to help older adults take right pills at the right time no matter where they are and when they need to. Practically, it is not easy to say which one among activities in ADL or IADL is more crucial than others, and its priority highly depends on the status of the target patient. The main reason of selecting ‘medication compliance’ as the goal of our prototype service is that taking medication appropriately is a common issue for normal people as well as older adults (for example, forget to take a pill due to participating in a meeting), and this could avoid that health condition is getting worse. A survey research shows that about 25 percent of American patients forget or ignore to take medications, leading them to pay higher healthcare cost or even needless deaths [70]. Accordingly, in this paper, we build a service that can help normal or old people keep their medication routine with the help of reminder and alerts through IoT products including wristwatch, smartphone, and home appliances such as lights and speakers.

### 5.2. Service Development

**Figure 14:**
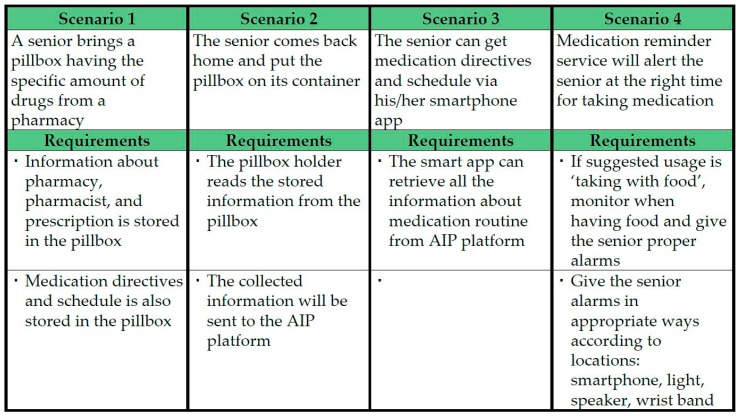
Service Scenario and requirements for aging in place (AIP) service platform.

We have summarized the service scenarios and requirements that allow seniors to be able to take their dose of pills at the right time as shown in Figure 14. We imagine a pharmacy prepared with an NFC (near field communication) tag writer to write data (e.g., prescription and usage) to the NFC tag embedded into a smart pillbox. Also, we imagine a senior’s home prepared with a pillbox holder equipped with an NFC reader.

**Figure 15:**
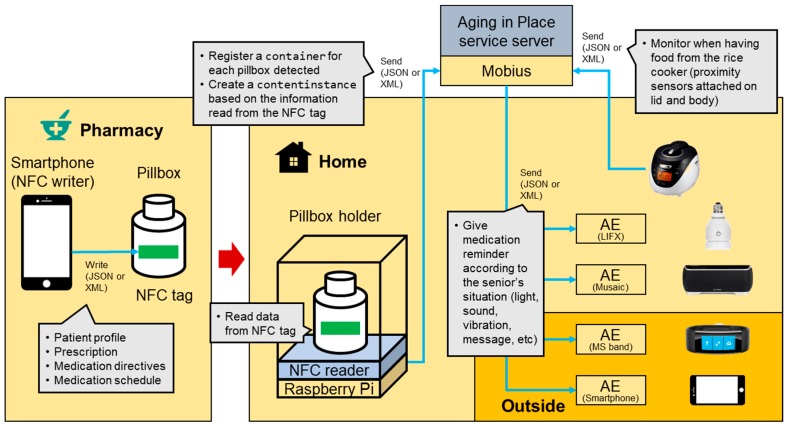
The overall diagram for the service scenario for medication reminder service using aging in place (AIP) service platform, smart pillbox kit, and various IoT products.

With the service scenario and requirements defined in Figure 14, Figure 15 illustrates the overall diagram for the service scenario for medication reminder service using AIP service platform, smart pillbox kit, and various IoT devices. From now on, we describe how to implement each part of the overall system.

#### 5.2.1. Pillbox and Pillbox Holder

**Figure 16:**
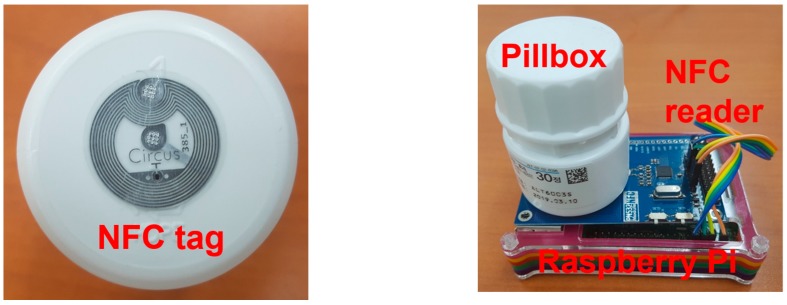
The pillbox with an NFC tag (**left**) and pillbox holder (**right**) consisting of a Raspberry Pi and NFC reader.

An off-the-shelf pillbox is created using a normal pillbox attached with a simple NFC tag, which contain various information about pharmacy, prescription, medication directives and schedule. A pillbox holder consisting of a Raspberry Pi and NFC reader is created, which will read data from the NFC tag attached into the pillbox, and send it to the AIP service platform. Figure 16 shows the pillbox and pillbox holder implemented.

**Figure 17:**
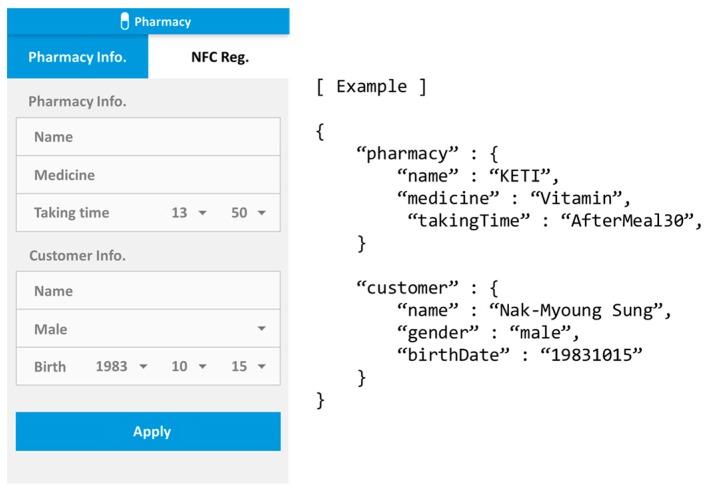
The smartphone app for pharmacy (**left**) and JSON format example (**right**).

For the pillbox kit, we have first developed a smartphone application for pharmacy, which is used to store data about patient, pharmacy, pharmacist, and medication in the NFC tag of the pillbox. Figure 17 shows a captured image of the app to input information and its JSON format example. We have also developed a smartphone app for seniors, which will be able to give medication reminder message to them at the right time according to medication schedule.

When the senior comes back home and puts the pillbox on the holder, the pillbox holder reads the data stored into the NFC tag of the pillbox and uploads it to the AIP service platform by creating a container resource for the pillbox and contentInstance resource for the information read from the tag, respectively, as shown in Figure 15.

#### 5.2.2. Sensors

**Figure 18:**
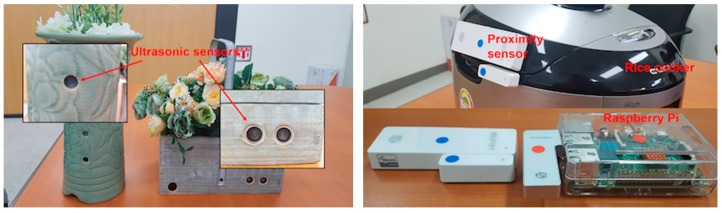
Ultrasonic sensors (**left**) and rice cooker embedded with proximity sensors (**right**).

Several ultrasonic sensors are developed and connected with a Raspberry Pi as shown on the left side of Figure 18. They could be installed inside a senior’s home, which detect the movement from room to room and identify the place the senior is located inside a home. Such information will be used to give the senior medication reminder in an appropriate way when it is time for medication.

If a dose of pills should be taken with food, it needs to detect whether or not the senior had food. To this end, we have attached a pair of proximity sensors onto the lid and body of a commercial rice cooker as shown on the right side of Figure 18. By using the proximity data between the cooker’s lid and body, we will be able to infer that the senior has opened the rice cooker and thus had food. Here, we assume that most Korean adults prefer a meal with a bowl of rice for their health.

#### 5.2.3. Actuators

**Figure 19:**
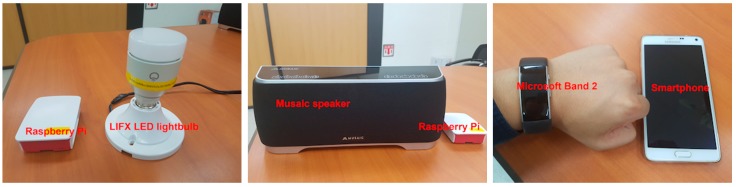
LIFX LED lightbulb (**left**), Musaic streaming speaker (**middle**), and Microsoft Band 2 (**right**).

We have deployed three types of actuators for our medication alarm service: LIFX, Musaic, and Microsoft Band 2, as shown in Figure 19. LIFX is an LED light bulb controlled remotely with a smartphone app via WiFi connection. Musaic is a speaker that can stream music in HiFi quality. Microsoft Band 2 is a wrist band can collect a variety of physical data from the body, for example, acceleration of body movement, skin temperature, heart rate, and sleep quality. Unfortunately, Microsoft no longer has sold the band since 2016 and currently no plan on releasing new band. However, we have adopted the Microsoft band for our AIP service because it is supported by a fully functional set of application programming interfaces (APIs) that allow developers to build their own band applications like vibration alarm.

LIFX and Musaic are IoT products complied with an IoT standard in the application layer, called AllJoyn, and thus we have implemented an interworking proxy entity on a Raspberry Pi that could translate protocol message each other between oneM2M and AllJoyn standards [71]. For Microsoft band we have development an interworking application in the smartphone connected with the band via Bluetooth connection.

Accordingly, all the actuator devices including LIFX, Musaic, and Microsoft Band 2 will be able to be connected with the AIP service platform and controlled by a CVO built for AIP service when it is time to take medication appropriately according to the senior’s context, i.e., location or physical status.

#### 5.2.4. Mobius and Interworking Proxy Entities (IPEs)

All the devices including the smart pillbox holder, sensors, and actuators described above are registered as application entities (AEs) in Mobius (i.e., oneM2M server platform), and their functions are mapped into corresponding containers each. Under each container a list of contentInstances are placed, each of which is associated with data or control message of the IoT products. Accordingly, all data or control commands for devices are hierarchically placed under their AEs, i.e., AE→container→contentInstance. Table 1 summarizes the AE, container, contentInstance for all the devices we have adopted for our AIP service. For example, in case of the pillbox holder, when the pillbox is placed on the pillbox holder, the data written in the NFC tag of the pillbox would be mapped into the contentInstance under the pillbottle01 container under the ketipillbox AE, which is information entered in the pharmacy’s smartphone app as shown in Figure 17.

All functions of each device are represented in the form of uniform resource identifiers (URIs), for example in case of the pillbox holder/ketipillbox/pillbottle01/latest. They would be able to be accessed via hypertext transfer protocol (HTTP) verbs (GET, PUT, POST, DELETE). Also, using the feature of subscription and notification of the Mobius platform, AEs could check whether or not any typical resource is updated and perform actions immediately and accordingly.

#### 5.2.5. Aging in Place (AIP) Service Platform

**Figure 20:**
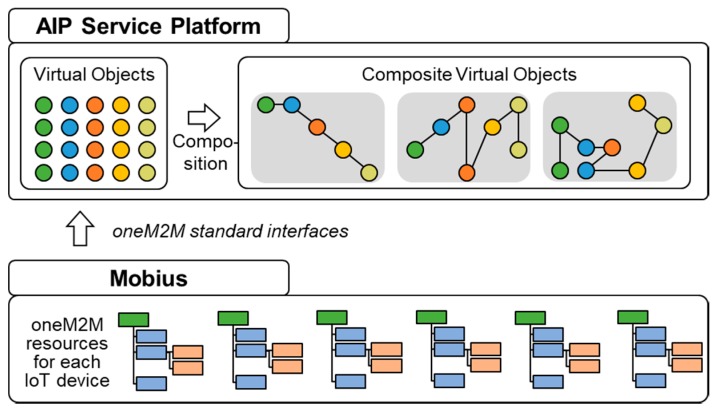
Procedure of converting oneM2M resources of IoT devices to virtual objects (VOs) and then composing composite virtual objects (CVOs) based on them.

Based on the resources of the chosen IoT devices (listed in Table 1) created in Mobius, AIP service platform creates the corresponding VOs and then we can create a CVO using the service composition tool for the medication reminder service as shown in Figure 20.

All the functions defined in Table 1 would be located in the form of the resources defined in the oneM2M standard inside the Mobius platform. Subsequently, the AIP service platform automatically converts each resource into the corresponding VO by discovering its semantic ontology located under the resource, as explained in Section 4.1. The VOs could be combined in a wide variety of ways to create a CVO, which is intended to perform a specific AIP service.

**Figure 21:**
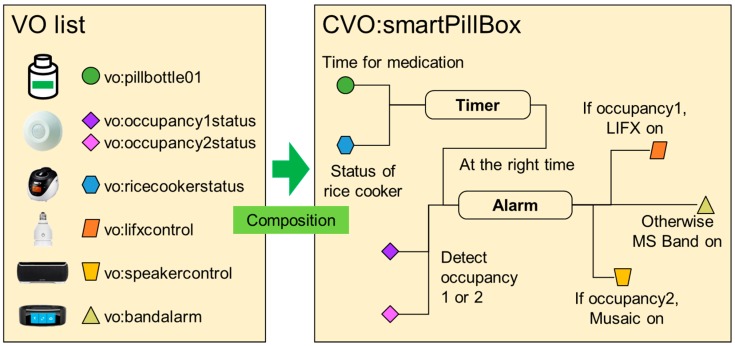
An example of CVO composition for a medication reminder service.

Figure 21 illustrates an example of CVO for our prototype service, medication reminder system. Here, we assume a senior needs to take a dose of pills with food. In the figure, CVO:smartPillBox consists of the following successive operations of VOs:

- Medication directives and schedule are retrieved from VO:pillbottle01,
- ‘Timer’ block calculates the times and conditions (e.g., within 30 minutes after having food) for medication and trigger ‘Alarm’ block at the right time when needed to take pills,
- According to the senior’s location (i.e., inferred from the occupancy status data, VO:occupancy1status, VO:occupancy2status), give medication reminder to the senior via VO:lifxcontrol (i.e., LIFX LED lightbulb) or VO:speakercontrol (i.e., Musaic speaker sound streaming), or otherwise VO:bandalarm (Microsoft Band 2 vibration).

In this way, any heterogeneous IoT products could be connected with Mobius using the proper interworking proxy entities and their resources are semantically represented in a standard form, and then we will be able to create a large variety of intelligent AIP services by composing CVOs based on the VOs of IoT products in an efficient way [72,73].

## 6. Limitations and Remaining Challenges

We have explained an efficient way of building services for aging in place using standard-based IoT platforms and heterogeneous IoT products. However, there might be additional practical issues we have to consider.

### 6.1. Combining Resource Oriented Architecture and Service Oriented Arhictecture

In the platform architecture we propose, an IoT device is registered as resources. For example, when a temperature sensor is registered, its name, function, and value of data type are created each in the platform, which could be identified and accessed through their URIs. This is called resource-oriented architecture (ROA) and has widely adopted for various standard IoT platforms.

However, the concept of service composition is driven from service-oriented architecture (SOA) whose atomic level is represented as an ‘atomic service’ per se. For example, a temperature sensor may be represented as a service for collecting temperature value. Accordingly, to allow for dynamic composition of IoT services it needs to combine the concept of ROA and SOA in a collaborative way.

Although we propose a method for combining ROA- and SOA-based systems using semantic technologies, the problem of resource access to make atomic services should be resolved because IoT devices are registered as different separated resources in standard IoT platforms.

For example, in order to compose a service for collecting temperature value with an IoT device having temperature sensors, we need to first discover a name resource matched with ‘temperature sensor’ and then discover a function resource matched with ‘collecting temperature value.’ This style of discovery and access to resources would be required much more frequently as the complexity involved in service composition increases, raising some technical challenges we need to address.

### 6.2. Service Composition Based on Semantic Interworking

Another important issue is to manage OWL-S when we compose services using ontologies. The OWL-S is designed for describing ontology-based services as mentioned in Section 4.1. It enables to automatically discover, invoke, compose, and monitor semantic-based resources. However, in practice, a service platform has its business logics with which a service needs to comply. Accordingly, in order to apply OWL-S to a service platform, it should support general business logics. Although we design CVOs based on modified OWL-S in our system, this could not support diverse business logics for different services. A common way to resolve this problem is to accommodate various different business logics. However, it is not efficient because we should understand the meaning of all of the logics and then select one logic appropriate for the requested service type. Consequently, we need to consider a more efficient way for applying OWL-S to a service platform in order to support automatic service composition, which is our next challenging task.

Another issue is to consider employing special reasoners. In our work, we present two different types of ontologies including VO and CVO. The VO is modified from the oneM2M Base Ontology, which is designed to interwork with different types of ontologies such as RDF, OWL-lite, OWL-DL, and OWL-Full without any special semantic reasoners. This means that the VO is worked by SPARQL queries (i.e., not only with a reasoner). The CVO represents services in this work using modified OWL-S. It could be operated by the SPARQL queries. Also, the CVO is operated by software modules of the AIP service platform. However, in order to support all practical IoT services and their scenarios, we need to support a large number of queries. To address this issue, we could consider employing reasoners. However, employment of special semantic reasoners may not support shallow ontologies, and then will be another challenging task.

Finally, we compare our proposed solution with the FIESTA-IoT project introduced in Section 2. In the FIESTA-IoT platform, a top-level ontology is proposed, which has taken the IoT-lite ontology, SSN ontology, and M3-Lite taxonomy. In addition, the FIESTA-IoT platform allows to create dynamic IoT services using the resources of heterogeneous testbeds and different IoT platforms connected with the FIESTA-IoT platform. However, in this paper, we focused on standard-based IoT services. In the perspectives, we have two different points as (1) the proposed system does not collect any resource from testbeds and IoT platforms; (2) the proposed system enables to dynamically define service scenarios using the service composition tool. The first point explains that our proposed system discovers and executes IoT resources from the Mobius by interworking with other different systems whereas the FIESTA-IoT platform directly collects resources from testbeds and IoT platforms based on FIESTA-IoT ontology and then perform semantic discovery and execute services in the FIESTA-IoT platform. The second point explains that our proposed system can create service scenarios using a graphical interface without programming for applications in contrast to the FIESTA-IoT project.

### 6.3. Usability and Effectiveness of the Aging in Place Service Platform

Although the NFC-embedded pillbox and holder are used to integrate medication data into the AIP service platform, it would be somewhat impractical with no pharmacies being ready to use the NFC-enabled pillbox but also few people being equipped with NFC tag readers at home. However, we chose this ‘unrealistic’ scenario in order to show the capability of sharing AIP-related data (e.g., prescription and usage for medication) between all stakeholders related to the aging-in-place domain (i.e., medical professionals, caregivers, doctors, nurses, family members, etc.) through a standard IoT platform. To this end, we fictitiously imagined an NFC-enabled pill box reader and writer allowing for integrating medication data into the platform. Of course, we assumed that such wireless tag-embedded everyday objects could be instrumented around our residential spaces in the near future. Also, we envision that all kinds of health-related data would be integrated into our standard platform (provided the platform will be deployed in medical information systems) and thus utilized by a variety of healthcare and wellness services in a consistent way across different hospitals and clinics.

Another important issue we need to address is conducting practical user studies. The best way to validate the performance of our platform and service composition tool is to recruit old adults and their families who are willing to participate into, and conduct user studies that can show the practical feasibility by analyzing how they use our systems. However, in this paper we have evaluated only the functional capabilities of the AIP platform and service composition tool by developing the chosen medication reminder scenario, but not adaptability and usability from the perspectives of users, i.e., older adults’ family members. Accordingly, for more practical validation of the proposed system, we plan to jointly work with European research projects related to IoT technologies development and experiments (our team has participated in), e.g., FIESTA-IoT (Federated Interoperable Semantic IoT Testbeds and Applications). We expect that it will be a good opportunity for us to test and validate our proposed system, which will be our future studies.

When building AIP services, the facility and software for constructing the services need to be modified from the perspectives of the end users. For example, in case of experiments with participants in other countries having a different lifestyle (e.g., Europe or U.S.), the rice cooker employed in our work to detect if older adults had food and infer their behaviors should be replaced with other types of home appliances appropriate for their everyday lives, for example, coffee pot, oven, microwave, or pressure cooker as illustrated in the previous research [46]. Also, the software allowing for defining rules for medication compliance need to be provided according to the extent of mental ability and level of understanding of technologies of end users. In our work, we assume that the end users would be medical professionals who provide and maintain AIP services or informal caregivers, including family members and friends, who have little knowledge on computing systems, but not older adults themselves. This implicates that the way of defining rules illustrated in Figure 10 might be not practical for seniors having mental disorder or cognitive impairment. However, if a user-friendly and more intuitive GUI (graphic user interface) based tool (as illustrated in our previously published literature for example TTEO [21] or IFTTT [6]) would be employed to organize rules, normal end users (i.e., older adults having normal mental abilities) could also define and modify services according to their preference and environmental context.

## 7. Conclusions

The growing elderly population is a worldwide issue. Many governments and local societies in the world are struggling with an increasing societal cost for public healthcare services. In particular, people living with old seniors having age-related diseases such as Alzheimer’s disease and dementia need to continuously monitor and help them perform activities in daily lives but also prevent them from being faced with emergency situations. Accordingly, to reduce the national and social burden of the healthcare for the elderly, a variety of research activities have been done under the name of aging in place technologies, which allow old adults to live independently in their current residence as long as possible. Our work proposed in this paper is as part of the effort to help old adults to stay and live in their home with minimal assistance requested for their everyday lives. With our system, any heterogeneous IoT products could be connected in a standardized way, and people could build a wide variety of services with the service composition tool for aging in place (but also for any other service domains) using the functionalities of the IoT products. We envision that the proposed method consisting of the aging-in-place service platform and service composition tool will be able to contribute to reduce the increasing healthcare cost for elderly people but also give informal caregivers including family members and friends peace of mind.

## Figures and Tables

**Table 1:** AE, container, contentInstance creation for IoT devices.

Device		AE Name	Container Name	Content Format (Contentinstance)	Standard
Pillbox Holder		Ketipillbox	Pillbottle01	JSON	oneM2M
Sensor	Ultrasonic sensor	occupancy1, occupancy2	status	Boolean “true” (true, false)	oneM2M
	Rice cooker	ketiricecooker	powerstatus	String “54 kwh”	Z-Wave
			openstatus	String “open” (open, close)	
Actuator	LIFX	ketilifx	lifxstatus	String “0,360” 0: on/off status, 360: color	AllJoyn
			lifxcontrol	String “power,1”,“hue,360” power,1: (0 = off, 1 = on) hue,360: (0~360 color)	
	Musaic	ketispeaker	speakercontrol	String “status,play” (play, stop, resume, next, previous, pause)	AllJoyn
	MS band	ketimsband	bandinfo	JSON	X
			bandalarm	String ”run” run: control command	
